# Photobiomodulation With Blue Laser Inhibits Bladder Cancer Progression

**DOI:** 10.3389/fonc.2021.701122

**Published:** 2021-10-18

**Authors:** Yuqi Xia, Weimin Yu, Fan Cheng, Ting Rao, Yuan Ruan, Run Yuan, Jinzhuo Ning, Xiangjun Zhou, Fangyou Lin, Di Zheng

**Affiliations:** Department of Urology, Renmin Hospital of Wuhan University, Wuhan, China

**Keywords:** bladder cancer, photobiomodulation, blue laser, epithelial-mesenchymal transition, MAPK pathway

## Abstract

Blue lasers are becoming more widely used in the diagnosis and treatment of bladder cancer; however, their photobiomodulation effects on bladder cancer cells remains unclear. The purpose of the current study was to explore the photobiomodulation effect of blue laser irradiation on bladder cancer progression and the associated mechanisms. The human uroepithelial cell line SV-HUC-1 and human bladder cancer cell lines T24 and EJ were exposed to blue laser irradiation (450 nm) at various energy densities, and cell proliferation, migration, invasion, epithelial-mesenchymal transition (EMT), and the levels of the proteins associated with the MAPK pathway proteins were determined. A significant decrease in cell viability was observed in a density-dependent manner after blue laser irradiation at > 4 J/cm^2^ in both bladder cancer cell lines. However, the blue laser did not reduce cell viability in SV-HUC-1 cells until the energy density exceeded 16 J/cm^2^. Meanwhile, Ki67 levels, reflecting cell proliferation and senescence, were also significantly decreased after blue laser irradiation at 4 J/cm^2^ and 8 J/cm^2^ in the absence of cell cycle arrest. Moreover, blue laser irradiation at 4 J/cm^2^ and 8 J/cm^2^ caused a reduction in cell migration and invasion and also reduced the expression levels of MMP-2, MMP-9, Snail, N-cadherin, phospho-MEK and phospho-ERK, and elevated the expression levels of E-cadherin. Meanwhile ERK activator(tBHQ) significantly reversed the irradiation-induced suppression of proliferation, migration and invasion in T24 and EJ cell lines. The present study showed that blue laser irradiation inhibited bladder cancer proliferation in a density-dependent manner and inhibited bladder cancer progression by suppressing migration, invasion, and the EMT process in T24 and EJ cell lines. This inhibition was possibly mediated *via* suppression of the MAPK/MEK/ERK pathway. Thus, the use of a low-energy blue laser in the diagnosis and treatment of bladder cancer is possibly safe and may have an anti-tumor effect.

## Introduction

Bladder cancer is the fourth leading cancer in males with an estimated 81,400 newly diagnosed cases in the USA in 2020 (1), and it remains the most common malignancy in the urinary system, with an expected 549,000 new cases and 200,000 deaths worldwide in 2018 (2, 3). Approximately 75% of bladder cancer cases are classified as non-muscle-invasive tumors (4). Although transurethral resection of bladder tumors and adjuvant therapy have been extensively used for the treatment of non-muscle invasive tumors, over 50% of the patients will eventually experience recurrence or progression (5, 6). Muscle invasive tumors account for 25% of bladder cancer cases; they require radical surgery or radiotherapy, which often leads to a significant decline in health-related quality of life and poor prognosis (7). Therefore, it is imperative to explore new methods for specific and sensitive diagnosis as well as effective treatment of bladder cancer. The diagnosis and treatment of bladder cancer traditionally depend on cystoscopy. To achieve early diagnosis, accurate staging, and complete tumor resection, numerous adjuvant methods have recently been developed by using noval techniques, including narrow-band imaging, photodynamic diagnosis (PDD), confocal laser endomicroscopy (CLE), and optical coherence tomography (8). The main principle of these methods is the use of low-energy laser light to obtain specific histopathological images (8).

In the past few years, various studies have demonstrated that low-level laser irradiation has an influence on gene expression and biological processes, including proliferation, apoptosis, and inflammation, which are known as photobiomodulation effects (9). Photobiomodulation has been widely applied in wound healing, pain relief, stem cell differentiation, and fibrosis inhibition in skin, oral, and osteogenic cells (10–12). However, the reported effects of photobiomodulation on cancer cells have been equivocal. Research has shown that laser irradiation at 780 nm (4 J/cm^2^) decreased cell proliferation and migration in oral squamous cell carcinoma (13). It has also been demonstrated that laser irradiation at 633 nm (120 J/cm^2^) induced apoptosis in lung adenocarcinoma cells (14). However, in another study, laser irradiation at 830 nm (1 J/cm^2^) stimulated the proliferation of head and neck carcinoma cells (15). These differences may be due to variations in cell type, wavelength, and irradiation parameters. The laser employed for PDD and CLE is in the range of the wavelengths of blue light (380–480 nm) (16). However, the photobiomodulation effects of blue laser on normal uroepithelial cells and bladder cancer cells have remained unclear despite its widespread use in the diagnosis and treatment of bladder cancer.

In this study, we evaluated the photobiomodulation effects of blue laser irradiation on the proliferation, migration, and invasion of normal uroepithelial cell line SV-HUC-1 and bladder cancer cell lines T24 and EJ. Additionally, we evaluated its effect on the expression of Ki67 and proteins associated with epithelial-mesenchymal transition (EMT) and potential signaling transduction.

## Materials and Methods

### Cell Culture

The human uroepithelial cell line SV-HUC-1 and human bladder cancer cell lines T24 and EJ were kindly provided by Stem Cell Bank, Chinese Academy of Sciences (Shanghai, China). SV-HUC-1 cells were cultured in F12K medium, and T24 and EJ cells were cultured in RPMI-1640 medium supplemented with 10% fetal bovine serum (FBS) (Gibco, USA) and 1% antibiotics (penicillin/streptomycin). The cells were maintained at 37°C in a humidified atmosphere with 5% CO_2_.

### Laser Irradiation

The cells were seeded in 6-well and 96-well plates. After 24 h, laser irradiation was conducted using a blue diode laser system (λ = 450 nm, Ligenesis, China) directly at the bottom of the well, as previously described (15), and each group used individual wells to prevent scattering. The distance between the plate and blue laser source was 13.5 cm to enable the light beam spot to cover all the cells. The cells were irradiated only once with a power density output of 100 mW/cm^2^ in the mode of continuous irradiation, and the exposure duration varied between 20 s and 240 s to achieve energy densities of 2 J/cm^2^, 4 J/cm^2^, 8 J/cm^2^, 12 J/cm^2^, 16 J/cm^2^, 20 J/cm^2^, and 24 J/cm^2^. Additionally, irradiation was performed in dark conditions to avoid the influence of other light. After irradiation, the medium was removed, and the cells were washed with phosphate buffered saline (PBS) twice to eliminate the influence of photo-induced molecular products in the medium. In the control group, the cells underwent the same operation without irradiation. To explore the potential signaling pathway, the activator of ERK, tert-Butylhydroquinone (t-BHQ, 50μM, HY-100489, MedChemExpress, USA) was used after laser irradiation at 8 J/cm^2^.

### Cell Proliferation Assay

Cell proliferation was detected using the Cell Counting Kit-8 (CCK-8) detection kit (Dojindo, Japan). Briefly, T24 and EJ cells were seeded in 96-well plates at a concentration of 2×10^3^ cells in 100 μl of medium per well. After 1 day, the cells were exposed to either no irradiation or blue laser for 2 J/cm^2^, 4 J/cm^2^, 8 J/cm^2^, 12 J/cm^2^, 16 J/cm^2^, 20 J/cm^2^, and 24 J/cm^2^. At time points of 24 and 48 h, 10 μl of CCK-8 reaction solution was added and the cells were further incubated for 1 h at 37°C. The absorbance of the samples was evaluated at 450 nm using an EnSight Multimode Plate Reader (Perkin Elmer, USA). The optical density (OD) values of each group relative to the control group represented the cell viability (%), and the values were used to estimate cell proliferation.

### Immunofluorescence Assay

Bladder cancer cells (1 × 10^6^) were plated in 6-well chamber slides and exposed to blue laser for 4 J/cm^2^, 8 J/cm^2^, or no irradiation. After 48 h, the cells were fixed with 4**%** paraformaldehyde for 30 min and incubated with blocking solution for 10 min at 20°C for permeabilization and non-specific antigen blocking. Next, the cells were incubated with rabbit anti-Ki67 primary antibody (1:150 dilution, 27309-1-AP, Proteintech, USA) overnight at 4°C. The cells were then incubated with goat anti-rabbit antibody for 50 min (AS-1109, ASPEN Biotechnology, China) as a secondary antibody, and the nuclei were stained with 4′,6-diamidino-2-phenylindole (AS1075, ASPEN Biotechnology, China). Images of five random fields were obtained for each group using a fluorescence microscope (BX51, Olympus, Japan). Ki67-positive cells were stained red in the nucleus. Five random fields were chosen from each slice, and the percentage of Ki67-positive cells was determined.

### Flow Cytometry

Apoptosis assays were performed using the FITC Annexin V Apoptosis Detection Kit I (BD, USA) following the recommended procedure. Cells were harvested 48 h after blue laser irradiation at 4 J/cm^2^, 8 J/cm^2^, or no irradiation and washed twice with PBS. Subsequently, cells were resuspended in 1X binding buffer and stained with Annexin-V-FITC and PI for 15min. Samples were analyzed by flow cytometry (Beckmancoulter, USA). The apoptosis cells were considered as the upper right quadrants.

Cell cycle assays were performed using the Cell Cycle Analysis Kit (KGA512, KeyGEN BioTECH, China) following the recommended procedure. Cells were harvested 48 h after blue laser irradiation at 4 J/cm^2^, 8 J/cm^2^, or no irradiation and fixed with 70% ethanol at 4°C overnight. Next, each sample was stained with working solution containing 450 μl propidium iodide and 50 μl RNase A for 30 min. A flow cytometer (FASC Calibur, BD, USA) was used to detect cell cycle distribution, and 15,000 events were measured for each sample.

### Migration and Transwell Invasion Assay

A scratch wound assay was performed to identify the migration capability of bladder cancer cells. Briefly, approximately 1 × 10^6^ bladder cancer cells were seeded in a 6-well plate and incubated overnight. A sterile 200 μl pipette tip was used to scratch the cell monolayers, after which the cell debris was removed by PBS and the serum-free medium was changed to inhibit cell proliferation. Next, blue laser irradiation at 4 J/cm^2^, 8 J/cm^2^, or no irradiation was applied, and the area of the wound was monitored at 0, 12, and 24 h using an inverted microscope (IX51, Olympus, Japan) at 40× magnification. Four random fields were photographed for each group, and the relative area of the wound was counted and compared with that of the controls.

Transwell migration and invasion assay was conducted to identify the migration and invasion capability of bladder cancer cells by using 24-well plates with(invasion) or without(migration) transwell chambers pre-coated with Matrigel matrix (1:8 dilution, 356234, BD, USA). After blue laser irradiation at 8 J/cm^2^ or no irradiation, 2 × 10^5^ bladder cancer cells were added to the upper chamber with serum-free medium, and the lower chamber contained medium with 20% serum as an attractant. After incubation for 48 h(invasion) or 24h(migration), the membranes were washed with PBS, fixed with 4% paraformaldehyde, and stained with 0.1% crystal violet (G1014, Servicebio, China). Finally, five random fields were imaged for each group using a microscope (IX51, Olympus, Japan) at 200× magnification, and the number of invaded cells was determined.

### Western Blotting Analysis

Forty-eight hours after blue laser irradiation at 4 J/cm^2^, 8 J/cm^2^ or no irradiation, bladder cancer cell proteins were extracted and quantified. Equivalent proteins were subjected to electrophoretic separation on a sodium dodecyl sulfate-polyacrylamide gel (SDS-PAGE) and then transferred to a polyvinylidene difluoride membrane (Millipore, Billerica, MS). The membranes were blocked with 5% skim milk and incubated with primary antibodies at 4°C overnight: Ki67(1:1000 dilution, 27309-1-AP, Proteintech, USA), CDK4(1:1000 dilution, #12790, Cell Signaling Technology, USA), MMP-2 (1:1000 dilution, ab92536, Abcam, UK), MMP-9 (1:500 dilution, ab76003, Abcam, UK), Snail (1:1000 dilution, #3879, Cell Signaling Technology, USA), E-cadherin (1:1000 dilution, 20874-1-AP, Proteintech, USA), N-cadherin (1:1000 dilution, 22018-1-AP, Proteintech, USA), MEK (1:1000 dilution, # 4694, Cell Signaling Technology, USA), p-MEK (1:1000 dilution, # 9154, Cell Signaling Technology, USA), ERK (1:2000 dilution, #4695, Cell Signaling Technology, USA), p-ERK (1:1000 dilution, #4370, Cell Signaling Technology, USA), p38 (1:1000 dilution, # 9212, Cell Signaling Technology, USA), p-p38 (1:1000 dilution, # 4511, Cell Signaling Technology, USA), and GAPDH (1:10000 dilution, ab37168, Abcam, UK). After washing the membranes twice with TBST buffer, the membranes were incubated with secondary antibodies (1:10000 dilution, AS1107, ASPEN Biotechnology, China) for 1 h. Chemiluminescence was performed using an ECL system kit (Beyotime Biotechnology, Shanghai, China). Integrated densities were determined using the ImageJ software (Fiji, NIH, USA).

### Statistical Analysis

All data are presented as mean ± standard deviation. Statistical analysis was performed using SPSS version 19.0 (SPSS Inc., USA). The means were compared using one-way analysis of variance followed by the Student-Newman-Keuls test for the different groups. The half maximal inhibitory concentration (IC50) was calculated using Graphpad Prism software. Differences were considered significant at *p* < 0.05. All experiments were performed at least three times.

## Results

### Blue Laser Inhibits the Proliferation of Bladder Cancer Cells

**Figure 1:**
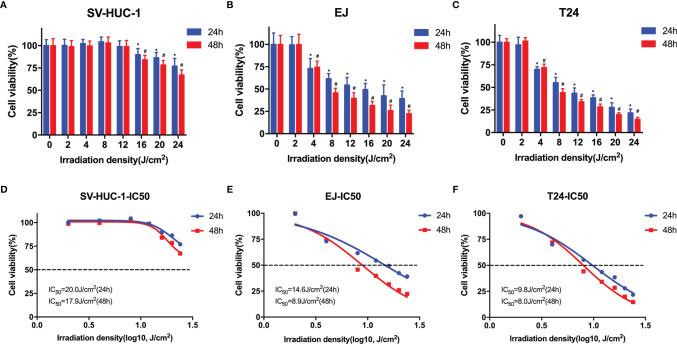
Blue laser inhibits the proliferation of bladder cancer cells. CCK-8 assay was performed to determine the effects of blue laser irradiation at 0 J/cm^2^, 2 J/cm^2^, 4 J/cm^2^, 8 J/cm^2^, 12 J/cm^2^, 16 J/cm^2^, 20 J/cm^2^, and 24 J/cm^2^ in SV-Huc-1 **(A)**, EJ **(B),** and T24 **(C)** cell lines 24 and 48 h following exposure. The IC_50_ curve of SV-Huc-1 **(D)**, EJ **(E),** and T24 **(F)** cell lines 24 and 48 h following exposure. ^*^ *p* < 0.05 *vs*. 0 J/cm^2^ group (24 h); ^#^ *p* < 0.05 *vs*. 0 J/cm^2^ group (48 h).

**Figure 2:**
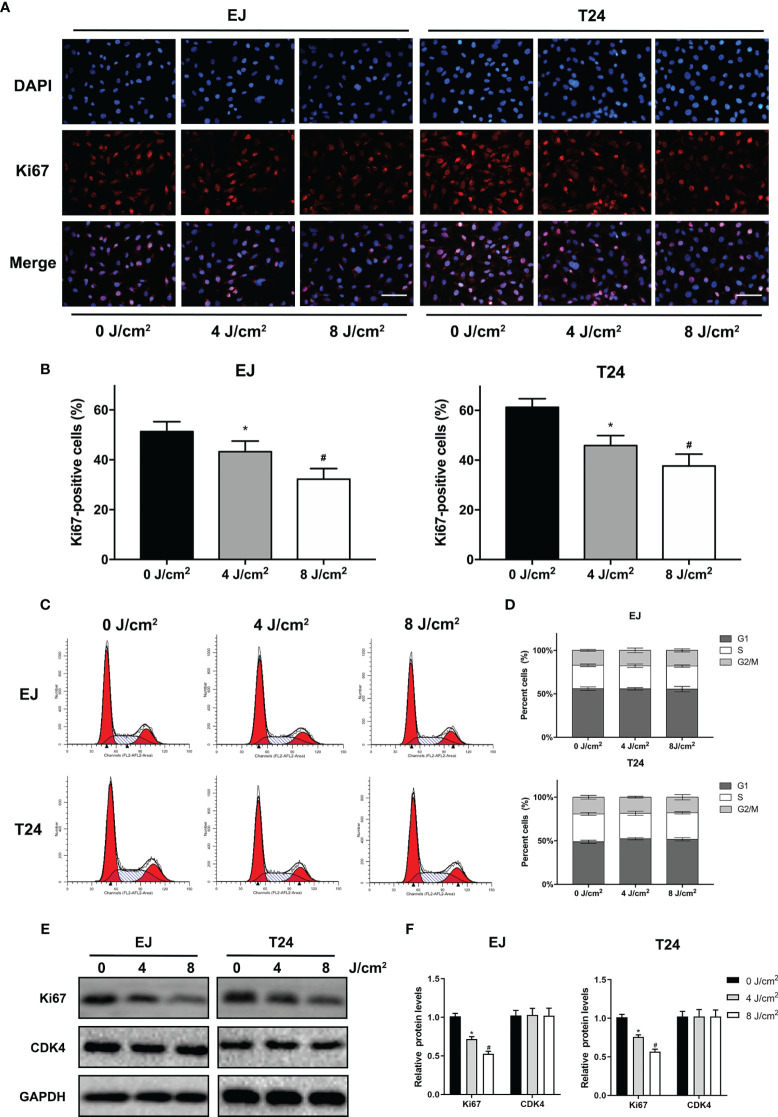
Blue laser inhibits proliferation by decreasing Ki67 levels without cell cycle arrest in bladder cancer cells. T24 and EJ cells were exposed to blue laser at 0 J/cm^2^, 4 J/cm^2^, and 8 J/cm^2^. **(A)** Representative images of Ki67 immunofluorescence assay (× 400, Scale Bar=100μm). **(B)** Determination of the percentage of Ki67-positive cells. **(C)** Representative figures of cell cycle analysis. **(D)** Quantification of cell cycle distribution. **(E)** Expression of Ki67 and CDK4 was detected by Western blot. **(F)** Quantification of relative expression levels of Ki67 and CDK4. ^*^ *p* < 0.05 *vs.* 0 J/cm^2^ group; ^#^ *p*< 0.05 *vs*. 4 J/cm^2^ group.

To determine the effects of blue laser irradiation on the proliferation of human uroepithelial cells and bladder cancer cells, a CCK-8 assay was performed. The human uroepithelial cell line SV-HUC-1 was used as a normal bladder cell line. Two well-studied types of bladder cancer cell lines, transitional cell carcinoma T24 and EJ cells, were used to increase the confidence level. Cells were exposed to blue laser at 0 J/cm^2^, 2 J/cm^2^, 4 J/cm^2^, 8 J/cm^2^, 12 J/cm^2^, 16 J/cm^2^, 20 J/cm^2^, and 24 J/cm^2^, and cell viability was evaluated after 24 and 48 h. The blue laser did not reduce the viability of SV-HUC-1 cells until the energy density was > 16 J/cm^2^ (**Figure 1A**). Compared with T24 and EJ cells, blue laser irradiation had a minimal impact on the viability of SV-HUC-1 cells. Cell viability was considerably lower than that in the control group upon irradiation with blue laser at > 4 J/cm^2^ in both bladder cancer cell lines at two time points in an energy density-dependent manner (**Figures 1B, C**). Meanwhile, 48h after blue laser irradiation, the IC_50_ of SV-HUC-1, EJ and T24 are 17.9J/cm^2^,8.9 J/cm^2^ and 8.0 J/cm^2^ respectively (**Figures 1D–F**). Moreover, the levels of Ki67, which reflects cell proliferation, were estimated by immunofluorescence assay and western blotting. As shown in **Figures 2A, B, E, F**, the protein expression of Ki67 were significantly decreased in T24 and EJ cells 48 h after irradiation with blue laser at 4 J/cm^2^ and 8 J/cm^2^. However, no significant changes in apoptosis rate (**Supplementary Figure 1**), cell cycle distribution, and CDK4(Cyclin-dependent kinase 4) expression (**Figures 2C, D**) were observed in either cell line. These findings suggest that blue laser irradiation could inhibit bladder cancer cell proliferation in the absence of cell cycle arrest.

### Blue Laser Inhibits Bladder Cancer Cell Migration and Invasion

**Figure 3:**
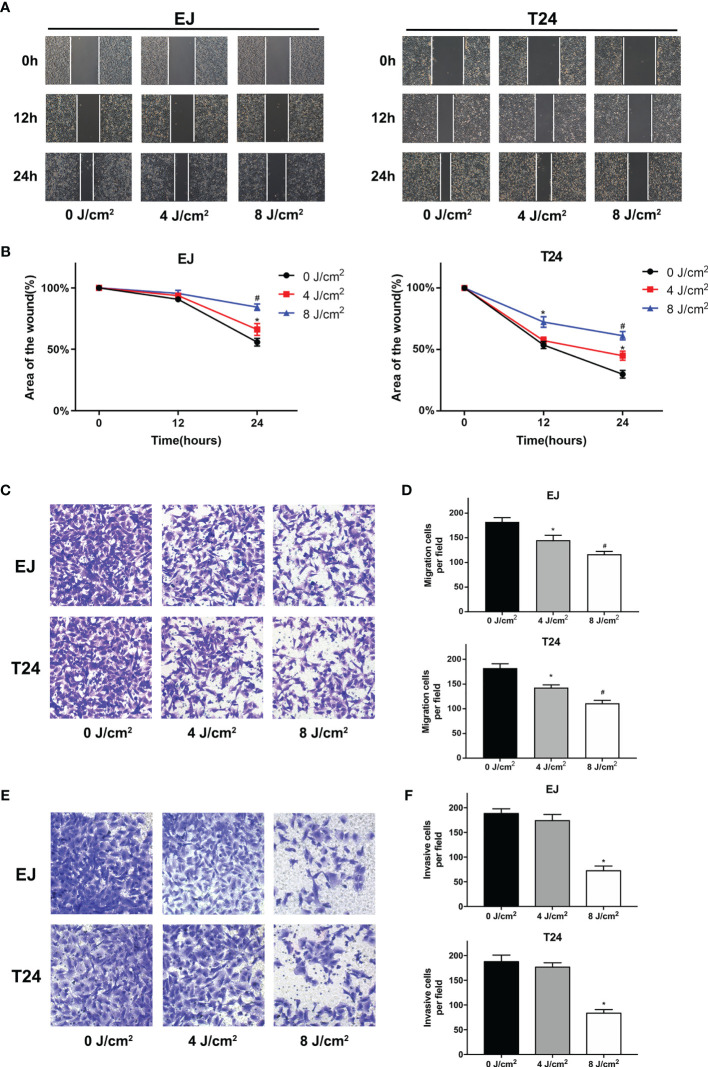
Blue laser inhibits the migration and invasion of bladder cancer cells. T24 and EJ cells were exposed to blue laser at 0 J/cm^2^, 4 J/cm^2^, and 8 J/cm^2^. **(A)** Representative images of scratch-wound assay pictured at 0, 12, and 24 h (×40). **(B)** The relative area of the wound at 0, 12, and 24 h **(C)** Representative images of transwell migration assay pictured 24 h following exposure (× 200). **(D)** Quantification of the number of migration cells per field. **(E)** Representative images of transwell invasion assay pictured 48 h following exposure (× 200). **(F)** Quantification of the number of invasive cells per field. ^*^ *p* < 0.05 *vs*. 0 J/cm^2^ group; ^#^ *p*< 0.05 *vs*. 4 J/cm^2^ group.

**Figure 4:**
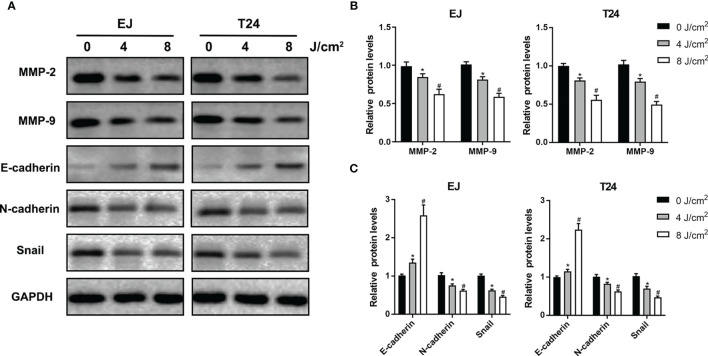
Blue laser inhibits invasion and EMT-associated proteins in bladder cancer cells. T24 and EJ cells were exposed to blue laser at 0 J/cm^2^, 4 J/cm^2^ and 8 J/cm^2^. **(A)** Expression of MMP-2, MMP-9, E-cadherin, N-cadherin and Snail proteins was detected by western blotting. **(B)** Quantification of relative expression levels of MMP-2 and MMP-9. **(C)** Quantification of relative expression levels of E-cadherin, N-cadherin and Snail. ^*^ *p* < 0.05 *vs*. 0 J/cm^2^ group; ^#^ *p*< 0.05 *vs*. 4 J/cm^2^ group.

Cell migration and invasion play crucial roles in the pathophysiology of cancer progression. To identify the effects of blue laser irradiation on the migration and invasion of bladder cancer cells, scratch wound, transwell migration and invasion assays were performed. As shown in **Figures 3A–D**, blue laser irradiation at 4 J/cm^2^ and 8 J/cm^2^ resulted in a significant reduction in cell migration in both T24 and EJ cell lines 24 h after treatment. Next, the transwell invasion assay was performed to further estimate invasion capability. Blue laser irradiation at 8 J/cm^2^ notably repressed bladder cancer cell invasion 48 h after irradiation, compared with the control treatment (**Figures 3E, F**). To further explore the potential mechanism, western blotting was employed to detect the expression levels of invasion-associated proteins (MMP-2 and MMP-9) after blue laser irradiation at 0 J/cm^2^, 4 J/cm^2^ and 8 J/cm^2^. As expected, a decrease in the expression levels of MMP-2 and MMP-9 proteins was observed in cells exposed to blue laser at 4 J/cm^2^ and 8 J/cm^2^, compared with the control cells (**Figures 4A, B**). These findings revealed that blue laser irradiation significantly repressed the migration and invasion of bladder cancer cells.

### Blue Laser Inhibits the EMT Process in Bladder Cancer Cells

EMT is an essential process in the early stages of cancer metastasis. To further investigate the effects of blue laser irradiation on the EMT process, western blotting was performed to determine the expression levels of EMT-associated proteins in T24 and EJ cell lines. As shown in **Figures 4A, C**, compared with the control group, blue laser irradiation at 4 J/cm^2^ and 8 J/cm^2^ caused a remarkable decrease in the expression of the transcription factor Snail, which is a key molecule involved in the EMT process. In addition, blue laser irradiation significantly increased the protein expression of the epithelial marker E-cadherin and decreased the mesenchymal marker N-cadherin in both cell lines. These findings demonstrate that blue laser irradiation could inhibit the EMT process in bladder cancer cells.

### Blue Laser Inhibits the MAPK/MEK/ERK Signaling Pathways in Bladder Cancer Cells

**Figure 5:**
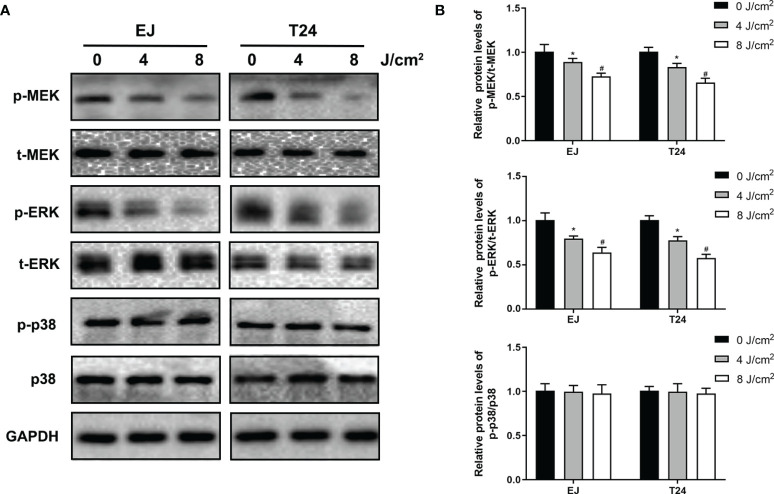
Blue laser inhibits the MAPK/MEK/ERK signaling pathways in bladder cancer cells. T24 and EJ cells were exposed to blue laser at 0 J/cm^2^, 4 J/cm^2^ and 8 J/cm^2^. **(A)** Expression of p-MEK, t-MEK, p-ERK, t-ERK, p-p38 and p38 proteins was detected by western blotting. **(B)** Quantification of relative expression levels of p-MEK/t-MEK, p-ERK/t-ERK, p-p38 and p38. ^*^ *p* < 0.05 *vs*. 0 J/cm^2^ group. ^#^ *p*< 0.05 *vs*. 4 J/cm^2^ group.

To investigate the potential mechanism associated with the anti-proliferation and anti-metastasis effects of blue laser irradiation, the relevant proteins in the MAPK pathway, mitogen-activated protein kinase kinase (MEK), extracellular signal regulator kinase (ERK) and p38 mitogen-activated protein kinase (p38) were detected by western blotting. As shown in **Figure 5A**, blue laser irradiation at 4 J/cm^2^ and 8 J/cm^2^ downregulated the expression levels of phospho-MEK(p-MEK) and phospho-ERK(p-ERK) in T24 and EJ cell lines. Nevertheless, no significant difference was detected between the three groups with regard to the expression levels of total MEK(t-MEK) and total ERK (t-ERK). Further quantitative analysis showed that the relative expression levels of p-MEK/t-MEK and p-ERK/t-ERK decreased after blue laser irradiation (**Figure 5B**). Meanwhile, no significant difference was detected in the relative expression levels of p-p38/p38 between the three groups.

**Figure 6:**
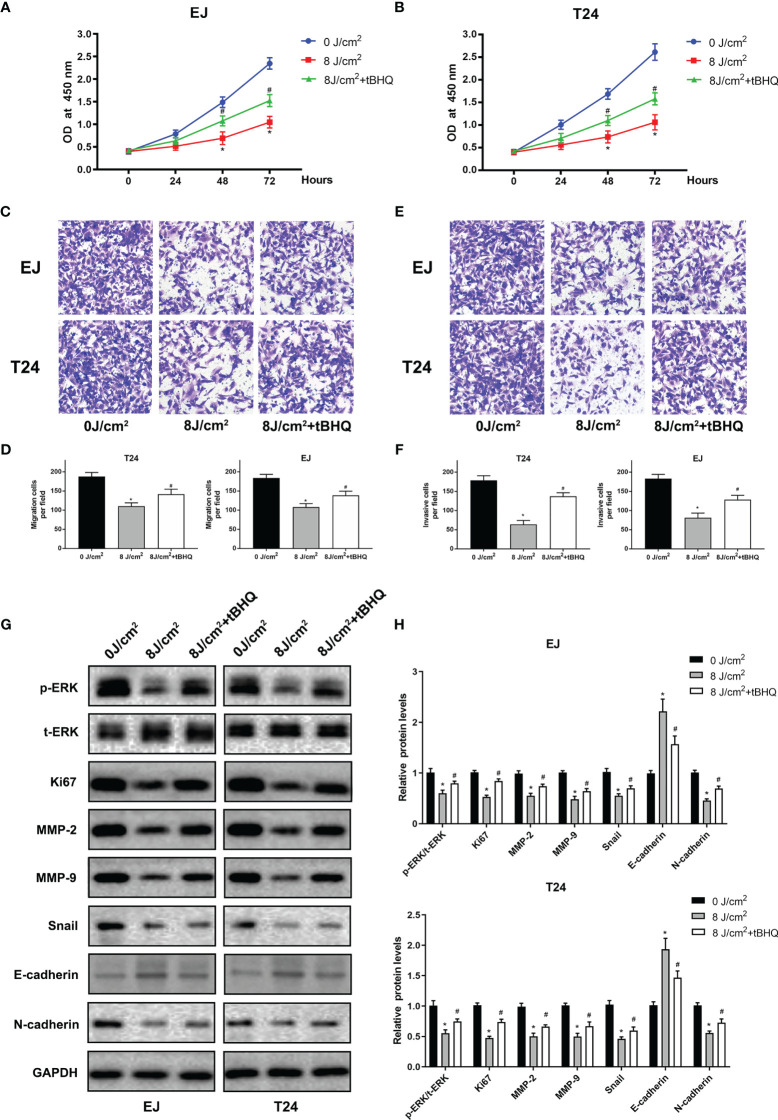
ERK activator(tBHQ) significantly reversed the irradiation-induced suppression of proliferation and migration in bladder cancer cells. CCK-8 assay was performed in EJ **(A),** and T24 **(B)** cell lines 24, 48 and 72 h following exposure. **(C)** Representative images of transwell migration assay pictured 24 h following exposure (× 200). **(D)** Quantification of the number of migration cells per field. **(E)** Representative images of transwell invasion assay pictured 48 h following exposure (× 200). **(F)** Quantification of the number of invasive cells per field. **(G)** Expression of p-ERK, t-ERK, Ki67, MMP-2, MMP-9, E-cadherin, N-cadherin and Snail proteins was detected by western blotting. **(H)** Quantification of relative expression levels of p-ERK/t-ERK, Ki67, MMP-2, MMP-9, E-cadherin, N-cadherin and Snail. ^*^ *p* < 0.05 *vs*. 0 J/cm^2^ group. ^#^ *p*< 0.05 *vs*. 8 J/cm^2^ group.

**Figure 7:**
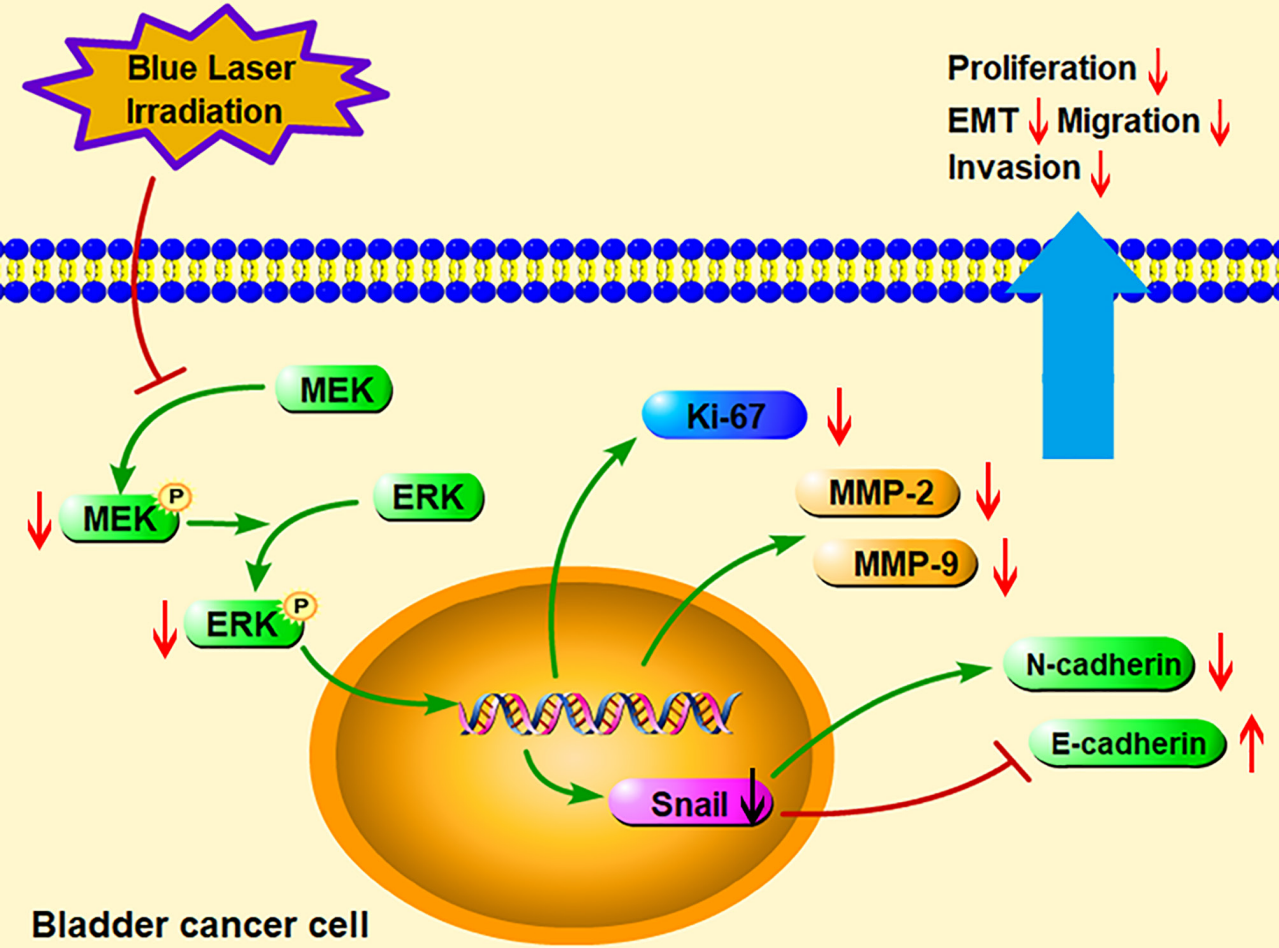
A schematic diagram illustrated the hypothesis of the present study. Blue laser irradiation inhibited the phosphorylation of MEK and ERK to regulate expression of the downstream signaling molecules. Furthermore, blue laser irradiation increased E-cadherin but decreased Ki67, MMP-2, MMP-9, Snail, and N-cadherin, consequently suppressed bladder cancer cell proliferation, migration, invasion, and EMT processes. EMT, Epithelial-mesenchymal transition; MEK, Mitogen-activated protein kinase kinase; ERK, Extracellular signal regulated kinase; MMP, Matrix metalloproteinase.

To further identify the role of MAPK/MEK/ERK pathway, we used the activator of ERK (tBHQ) after blue laser irradiation at 8 J/cm^2^ in proliferation, transwell migration and western blot assay. As shown in **Figures 6A–F**, tBHQ significantly reversed the irradiation induced suppression of proliferation, migration and invasion in T24 and EJ cell lines. Further, the western blot indicated that, the expression of p-ERK, Ki67, MMP-2, MMP-9, Snail, E-cadherin and N-cadherin were significantly reversed after treatment with the activator of ERK (tBHQ) compared with the 8 J/cm^2^ group (**Figures 6G, H**). Taken together, blue laser irradiation possibly inhibited the proliferation, migration, invasion, and EMT of bladder cancer cells through the MAPK/MEK/ERK signaling pathway (**Figure 7**).

## Discussion

Currently, new methods for specific and sensitive diagnosis cancer are wildly explored, including imaging techniques (8) and molecular markers (17). While the safety and efficacy of low-energy laser irradiation in patients with cancer have remained unclear. As earlier mentioned, laser irradiation stimulates the immune system or the production of excessive reactive oxygen species (ROS) to attack tumors under certain conditions (18); however, under other conditions, laser irradiation facilitates cancer growth (19). Thus, it is of great significance to explore the relationship between laser irradiation and cancer progression at various wavelengths, irradiation densities, and cancer types.

Despite that blue laser is becoming more widely used in the detection and treatment of bladder cancer, its photobiomodulation effect on bladder cancer cells has remained unclear. Several *in vivo* and *in vitro* studies have demonstrated that blue light irradiation exerts antimicrobial, anti-inflammatory, and anticancer effects on skin diseases and cancer, such as acne vulgaris (20), breast cancer (21), and colon cancer (22) by regulating miRNAs, ROS, and the mitogen-activated protein kinase (MAPK) signaling pathway. In the current study, we used a blue laser with a wavelength of 450 nm, which is widely applied in clinical practice (16). Meanwhile, different laser parameters often result in different effects on cellular metabolism; thus, we explored different energy densities based on previous studies (13, 14). We found that blue laser irradiation inhibited the proliferation of bladder cancer cells in a density-dependent manner. As the energy density increased, the photobiomodulation effect of the laser became enhanced. Furthermore, compared with T24 and EJ cells, blue laser irradiation had a minimal impact on the viability of normal uroepithelial SV-HUC-1 cells until the energy density was > 16 J/cm^2^. We inferred that a high energy density > 16 J/cm^2^ may damage bladder cells.

Cell senescence, defined as the irreversible decline of proliferative capacity, is characterized by telomere shortening, chromatin remodeling, and mitochondrial alterations. The level of proliferation marker Ki67, one of the indicators of senescence, has a negative correlation with the senescent state of cells (23). We found that Ki67 levels significantly decreased after irradiation without cell cycle arrest, which was consistent with the results of a previous study (15). The results implied that blue laser irradiation inhibited the proliferation of bladder cancer cells, which may be associated with the induction of cell senescence.

Migration and invasion of peripheral tissues, which eventually leads to metastasis, is an essential biological behavior of cancer. Matrix metalloproteinases (MMPs) are extracellular matrix (ECM)-degrading enzymes that are involved in the invasion of tumor cells into the basement membrane (24). Therefore, it is an effective marker for predicting tumor progression. In this study, scratch-wound and transwell assays were conducted to estimate the migration and invasion capacity of cancer cells. We found that blue laser irradiation inhibited the migration and invasion of bladder cancer cells. Subsequently, western blotting showed that the protein expression of ECM-degrading enzymes MMP-2 and MMP-9 decreased after blue laser irradiation. EMT is an essential process in cancer metastasis. During EMT, epithelial cells detach and enter into a mesenchymal state and hence become motile and gain invasive capacity (25). EMT is initiated by EMT-inducing transcription factors Snail, Zeb, and Twist, which lead to the repression of epithelial state-associated genes, such as E-cadherin, and result in the activation of genes associated with the mesenchymal state, such as N-cadherin and vimentin (25). Thus, the expression levels of Snail, N-cadherin, and E-cadherin are usually used as indicators of the level of EMT. In the current study, the findings indicate that blue laser irradiation inhibited EMT through the suppression of Snail and N-cadherin and the upregulation of E-cadherin. Taken together, these results indicate that blue laser irradiation inhibits bladder cancer progression by suppressing migration, invasion, and the EMT process.

The signaling pathways involved in the inhibition of cancer progression by blue laser irradiation remain unknown. The MAPK/MEK/ERK signaling pathway consists of serine/threonine kinases that play a crucial role in cell proliferation, migration, and invasion, and EMT (26). In cancer cells, members of the MAPK family, including MEK/ERK, p38, and c-Jun N-terminal kinase (JNK), are phosphorylated and translocated from the cytoplasm to the nucleus to activate cell proliferation and regulate various downstream substrates, including MMP-2, MMP-9, and EMT-associated genes (27–29). Previous studies have demonstrated that the MAPK pathway mediates cancer progression under stress conditions, such as light irradiation. In an *in vitro* study, blue light irradiation induced the apoptosis of colon cancer cells through the suppression of ERK and the activation of JNK (30). Another study showed that blue light irradiation inhibited the migration and invasion of fibrosarcoma cancer cells by suppressing ERK (22). In addition, it has been reported that blue light irradiation induces oxidative stress and mitochondrial damage by upregulating JNK in human retinal epithelial cells and retinal ganglion cells (31, 32). Consistent with the results of previous studies, we found that the levels of p-MEK and p-ERK significantly decreased after blue laser irradiation in bladder cancer cells, meanwhile ERK activator(tBHQ) significantly reversed the irradiation-induced suppression of proliferation, migration and invasion in T24 and EJ cell lines. Hence, we speculated that the MAPK/MEK/ERK pathway may be involved in the inhibition of bladder cancer progression by blue laser irradiation.

This study has some limitations. For instance, we only evaluated the effects of photobiomodulation on two bladder cancer cell lines *in vitro*. The anti-tumor impact of blue laser on the progression of bladder cancer *in vivo* is still unclear. More studies are needed to explore different wavelengths and the detailed mechanism *in vitro* and *in vivo*.

In conclusion, the findings of the current study showed that blue laser irradiation inhibited the proliferation of bladder cancer cells in a density-dependent manner and inhibited bladder cancer progression by suppressing migration, invasion, and the EMT process. This inhibition was possibly mediated by a suppressed MAPK/MEK/ERK pathway. Consequently, the use of a low-energy blue laser in the diagnosis and treatment of bladder cancer is possibly safe and may have an anti-tumor effect. These findings provide a novel potential therapeutic strategy for bladder cancer.

## Data Availability Statement

The raw data supporting the conclusions of this article will be made available by the authors, without undue reservation.

## Author Contributions

FC, WY, and YX designed the study. YX, TR, YR, and RY performed the experiments and collected the data. JN, XZ, FL, and DZ analyzed the data. YX and WY wrote the article. All authors contributed to the article and approved the submitted version.

## Funding

This research was supported by grants from National Natural Science Foundation of China (81870471 and 81800617) and Science and Technology Major Project of Hubei Province(2019AEA170).

## Conflict of Interest

The authors declare that the research was conducted in the absence of any commercial or financial relationships that could be construed as a potential conflict of interest.

## Publisher’s Note

All claims expressed in this article are solely those of the authors and do not necessarily represent those of their affiliated organizations, or those of the publisher, the editors and the reviewers. Any product that may be evaluated in this article, or claim that may be made by its manufacturer, is not guaranteed or endorsed by the publisher.

## Supplementary Material

Supplementary Figure 1 Blue laser doesn’t induce apoptosis in bladder cancer cells. **(A)** Representative figures of flow cytometry apoptosis analysis. **(B)** Quantification of apoptosis rate.
