# Supplementary figures and images for: Photobiomodulation With Blue Laser Inhibits Bladder Cancer Progression
Source: Front Oncol. 2021 Oct 18;11:701122. doi: 10.3389/fonc.2021.701122 (PMC8558536; doi:10.3389/fonc.2021.701122)

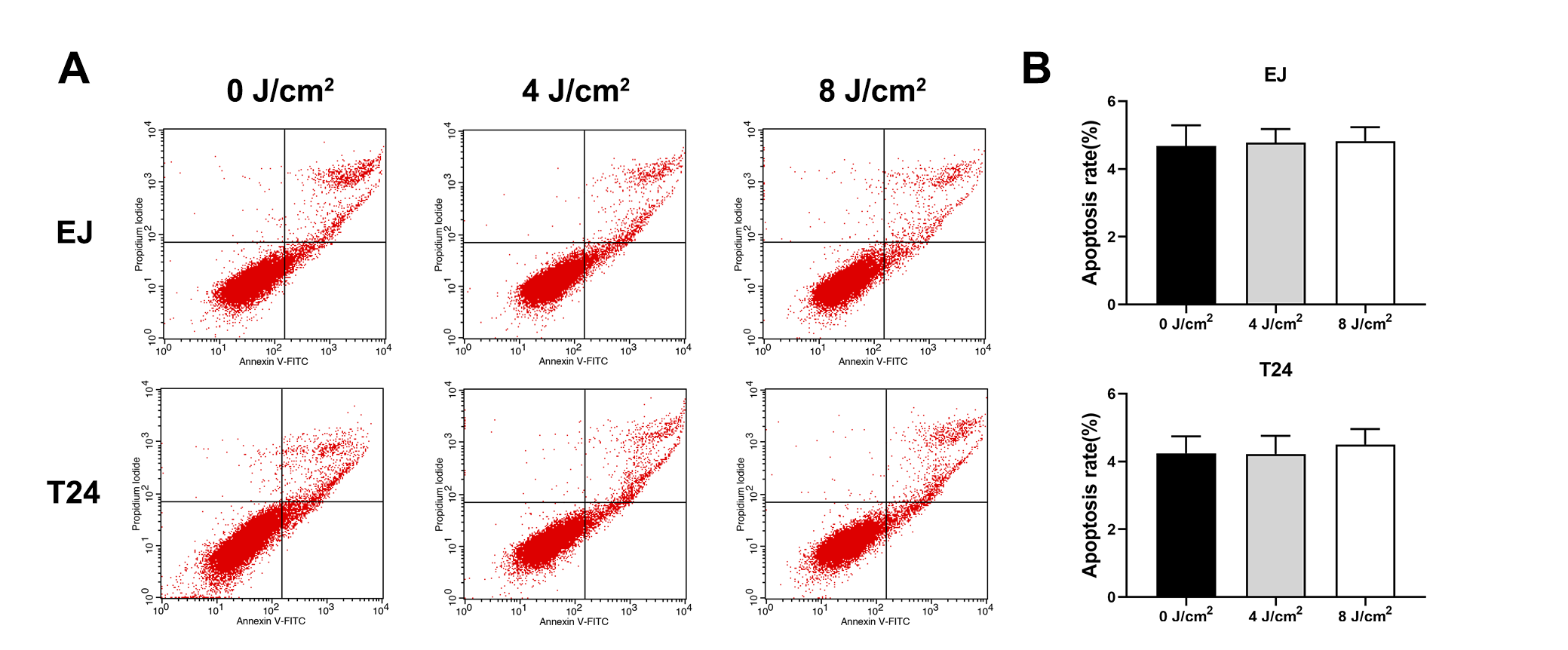

Supplement: Supplementary Figure 1 — Blue laser doesn’t induce apoptosis in bladder cancer cells. (A) Representative figures of flow cytometry apoptosis analysis. (B) Quantification of apoptosis rate. [file Image_1.tif]
